# Cortisol and Major Depressive Disorder—Translating Findings From Humans to Animal Models and Back

**DOI:** 10.3389/fpsyt.2019.00974

**Published:** 2020-01-22

**Authors:** L. Sanjay Nandam, Matthew Brazel, Mei Zhou, Dhanisha J. Jhaveri

**Affiliations:** ^1^ Mental Health Unit, Prince Charles Hospital, Brisbane, QLD, Australia; ^2^ Department of Psychiatry, Royal Hobart Hospital, Hobart, TAS, Australia; ^3^ Mater Research Institute, The University of Queensland, Brisbane, QLD, Australia; ^4^ Queensland Brain Institute, The University of Queensland, Brisbane, QLD, Australia

**Keywords:** major depressive disorder, cortisol, stress, antidepressants, preclinical models, behavior

## Abstract

Major depressive disorder (MDD) is a global problem for which current pharmacotherapies are not completely effective. Hypothalamic–pituitary–adrenal (HPA) axis dysfunction has long been associated with MDD; however, the value of assessing cortisol as a biological benchmark of the pathophysiology or treatment of MDD is still debated. In this review, we critically evaluate the relationship between HPA axis dysfunction and cortisol level in relation to MDD subtype, stress, gender and treatment regime, as well as in rodent models. We find that an elevated cortisol response to stress is associated with acute and severe, but not mild or atypical, forms of MDD. Furthermore, the increased incidence of MDD in females is associated with greater cortisol response variability rather than higher baseline levels of cortisol. Despite almost all current MDD treatments influencing cortisol levels, we could find no convincing relationship between cortisol level and therapeutic response in either a clinical or preclinical setting. Thus, we argue that the absolute level of cortisol is unreliable for predicting the efficacy of antidepressant treatment. We propose that future preclinical models should reliably produce exaggerated HPA axis responses to acute or chronic stress *a priori*, which may, or may not, alter baseline cortisol levels, while also modelling the core symptoms of MDD that can be targeted for reversal. Combining genetic and environmental risk factors in such a model, together with the interrogation of the resultant molecular, cellular, and behavioral changes, promises a new mechanistic understanding of MDD and focused therapeutic strategies.

## Introduction

Major depressive disorder (MDD) is a complex, multifactorial, and heterogenous clinical syndrome that currently affects at least 120 million people worldwide and by 2030 will be the single highest contributor to the global burden of disease (1). Existing therapies are not efficacious for all patients and over the past five decades few, if any, truly novel treatments for MDD have emerged that go beyond the monoamine theory of depression first presented in the 1960s (2). Although there is growing evidence that multiple other neurotransmitter systems (3), inflammatory processes (4), and dysregulation of the hypothalamic–pituitary–adrenal (HPA) axis are involved in MDD, these insights have not yet led to new treatments due to our limited understanding of their molecular mechanisms (5).

Progress in therapeutics has been hindered by the inadequacy of preclinical animal models to fully recapitulate the heterogeneous nature of the human condition (5). To improve this, researchers have sought to develop models that maintain adequate face, predictive, etiological, and construct validity with MDD (3). Specifically, candidate models need to demonstrate similarity between the behavioral phenotype and clinical symptoms (face validity), reversal of behavior with known or proposed antidepressants (predictive validity), and behavior inducible by factors associated with human disease (etiological validity), as well as displaying putative MDD biomarkers (construct validity) (3). In meeting these criteria, a major goal of preclinical animal models has been to emulate MDD to provide a framework for investigating neurobiological cause and effect mechanisms beyond those available from clinical studies.

The seemingly causal relationship between stress and cortisol in MDD, first described in the 1950s (6), promised just such a mechanism that was both independent of the monoamine theory and potentially able to deliver new therapeutic approaches. Accordingly, rodent models using environmental and hormonal stressors, addressing etiological and construct validity, respectively, have been developed. Quantitative changes in behavioral phenotypes, particularly those recapitulating core symptoms of major depression such as increased anxiety, anhedonia, and cognitive deficits, have been used for face validity. Multiple drug studies have subsequently explored the predictive validity of these models for new and established antidepressants. Although this approach has provided some new biological insights and increased the knowledge base, it has not yet produced the needed therapeutic breakthroughs.

In this review, we critically evaluate the validity of modelling HPA dysregulation in preclinical studies as a key driver of MDD pathophysiology. We do this by contrasting animal data with acute and chronic clinical studies that have examined stress, MDD treatment, and cortisol levels. We conclude by discussing the advantages and disadvantages of the current rodent models of MDD and HPA dysregulation, and how these might be used in combination with genetic risk factors and modern neuroscience tools to probe the neural circuitry of MDD and, ultimately, improve its treatment.

### Cortisol and MDD

**Figure 1:**
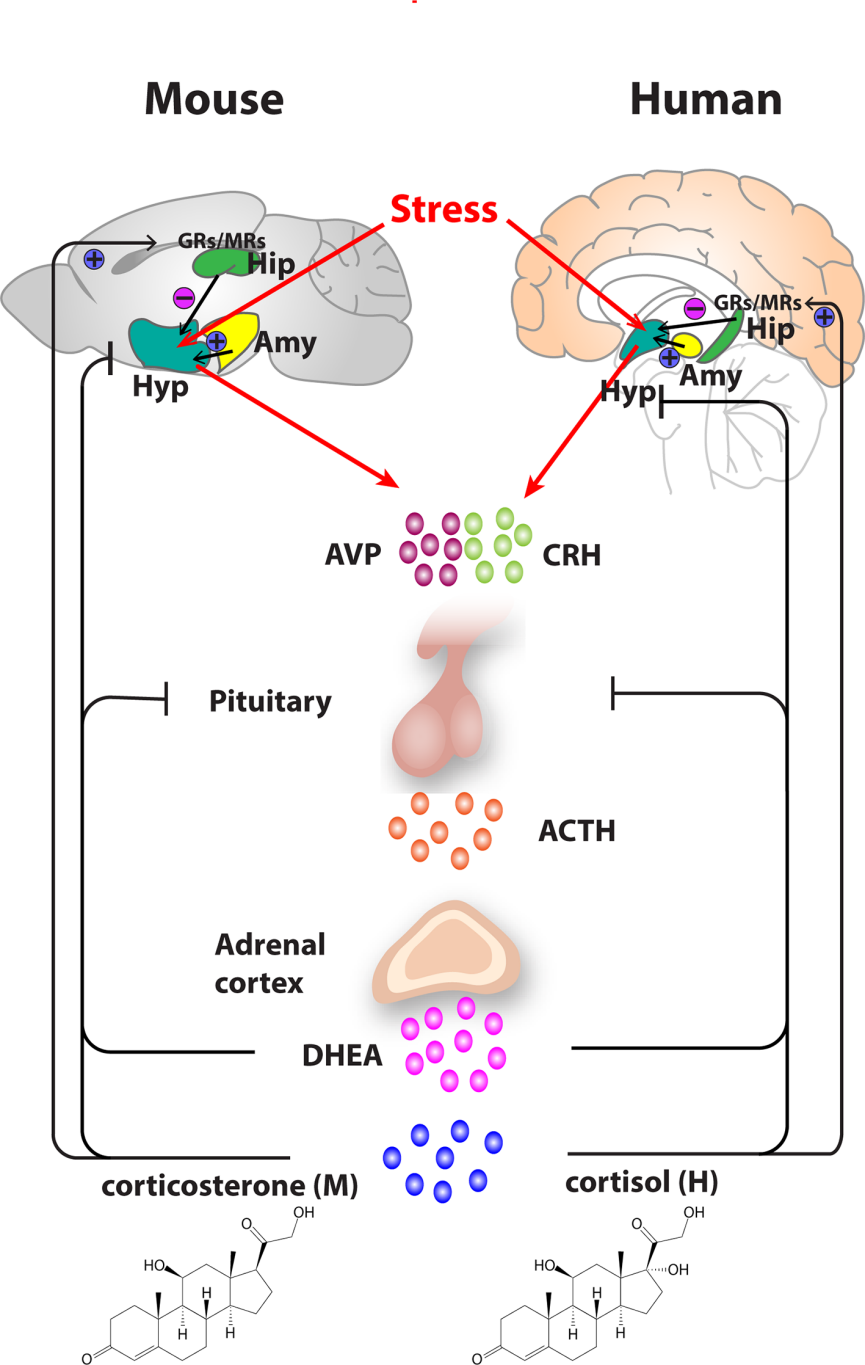
Organization of the hypothalamic–pituitary–adrenal (HPA) axis in mouse and human. In both mouse and humans, stress leads to secretion of corticotropin releasing hormone (CRH) and arginine vasopressin (AVP) from the hypothalamus, which subsequently stimulates the secretion of adrenocorticotropic hormone (ACTH) from the pituitary gland. ACTH transported *via* blood to the adrenal cortex of the adrenal gland stimulates the synthesis and secretion of glucocorticoids—primarily corticosterone, in mice and cortisol in humans as well as secretion of other major steroid dehydroepiandrosterone (DHEA). Glucocorticoids regulate their own secretion *via* a negative feedback control mechanism *via* mineralocorticoid (MR) and glucocorticoid receptors (GR) at the level of the hippocampus, hypothalamus, and pituitary gland. MRs, which have a high affinity for endogenous glucocorticoids, are found primarily in the hippocampus and determine the basal activity of the HPA axis. In contrast, the activation GRs upon binding of glucocorticoids in the hippocampus, hypothalamus, and pituitary leads to an inhibitory feedback onto the hypothalamus to control the stress response. A facilitatory input from amygdala to the hypothalamus also plays a role in the activation of the HPA axis response. Arrows represent stimulation, and T-shaped lines represent inhibition. Amy, Amygdala; Hip, Hippocampus; Hyp, Hypothalamus.

In MDD, glucocorticoid receptor (GR) signaling is abnormal (7) and associated with chronic hypersecretion from the corticotrophin releasing hormone (CRH) neurons of the HPA axis (**Figure 1**) (8). This hypersecretion is thought to shift HPA activity toward ever higher set points, resulting in the persistently elevated HPA activity seen in some MDD patients (9, 10). Many studies of MDD patients have reported abnormalities of cortisol suppression in response to pharmacological and psychological challenge (11–13), and probing the mechanisms underlying this effect has remained a popular line of inquiry for both clinical and translational research. However, the association between cortisol and MDD in humans is complex, and appears dependent on stage of illness, severity, and type of challenge employed. For example, with respect to stage of illness, HPA responsiveness does not appear to be affected in chronic MDD (symptoms of more than 2 years duration), with patients and controls not showing any difference in salivary and serum cortisol levels following dexamethasone suppression testing (DST), even after controlling for variability in dexamethasone metabolism (14). In contrast, remitted MDD patients generate higher cortisol levels than controls following exposure to a visual stress cue (15). In those with remitted MDD, persisting hyperresponsiveness to dexamethasone can predict relapse at 6 months (16), whereas cortisol levels do not provide prognostic information in chronic MDD (14).

With respect to the severity of MDD, a number of studies have shown that the severity of depressive symptoms is proportionate to cortisol level (16). More severe MDD subtypes, such as psychotic (17–20) and melancholic MDD (21, 22), consistently show higher baseline and challenge salivary and serum cortisol levels than observed in atypical MDD, a less severe MDD variant. Atypical MDD sufferers have been shown to have cortisol responses that are closer to those of healthy controls, especially when contrasted against the levels seen in melancholic MDD (21, 22). It has also been suggested that elevated cortisol may be responsible for the emergence of psychotic symptoms in severe MDD (23, 24). There are also marked HPA abnormalities in bipolar depression, which has a more severe presentation than milder forms of MDD (25). Elevated cortisol levels are also associated with the cognitive impairment seen in psychotic MDD (24).

Taken together, the evidence supports the notion that cortisol dysfunction is proportionate to, and consistent with, current nosological classifications of MDD severity. It appears that cortisol dysregulation, specifically in response to stress, is reliably associated with severe and acute presentations of MDD. Chronic and less severe subtypes of MDD, such as atypical depression, do not exhibit this robust association, which is consistent with data suggesting that personality and social factors may be major drivers of these subtypes (26). The association of HPA dysregulation with MDD severity might be mechanistically explained by parallel findings from receptor, immunological, and imaging studies. Melancholic and psychotic, but not atypical, depression show increased cortisol response to laboratory stress challenge (27). This may come about through altered functioning of GR expression, which then affects the homeostatic regulation of cortisol (28). The resultant hypercortisolism drives changes to serotonin receptors (29, 30), and this has been proposed as the mechanism underlying the specific symptoms of severe MDD (31). In atypical MDD, such symptoms are absent and hence serotonin receptor function is likely preserved (32). Elevated cortisol levels have also been proposed to contribute to the psychosis seen in psychotic MDD, *via* increased dopaminergic activity (33). Supporting this theory, mifepristone, a GR antagonist, has been shown to specifically reduce the psychotic symptoms of psychotic MDD (34, 35). Atypical MDD shows elevated inflammatory markers compared to melancholic MDD (22). Individuals with psychotic (36) and melancholic (37) MDD have been found to have smaller hippocampal volumes than those with less severe forms of depression. Frontal lobe volume is reduced in psychotic MDD (38), and the sylvian fissure is enlarged in melancholic MDD (39). In contrast to atypical depression, melancholic depression is characterized by reduced blood flow to the right frontal lobe, perhaps supporting the importance of social and personality in the former (40).

### Risk Factors for MDD and Cortisol Elevation

#### Early Life Stress, Cortisol, and MDD

Early life stress (ELS) programs for abnormal adult cortisol responses to stress challenge (41), potentially *via* glucocorticoid resistance, increased central CRH activity (42) and changes to GR and mineralocorticoid receptor (MR) binding (43). Abnormal cortisol responses persist into adulthood (44) and likely underpin the impaired HPA axis function seen in several trauma associated psychiatric disorders, including MDD. Nonetheless, the precise way that ELS affects adult HPA function appears complex. In an instructive study, for adolescents with a history of stressful life events, those who experienced recurrent depression exhibited a persistently elevated cortisol response to a laboratory stress task (32). In the same cohort, those who were currently experiencing their first episode of MDD exhibited a blunted cortisol response to the same task (32). Despite all the adolescents having had a prior history of ELS, there were no differences in baseline cortisol levels, and correlation with MDD was only demonstrated by variance in cortisol reactivity, rather than absolute level, to the task (32). This range of state and task dependent cortisol changes in a single cohort parallels the varied findings in the cortisol and ELS literature (45). Several studies report increased cortisol reactivity to stress in ELS populations (44, 46–49), whereas, contrary data showing reduced cortisol responses have also been reported (50–53). Variance in HPA axis outcome to ELS have been shown to be dependent on the timing of the stress (54). ELS from birth to age 5 years does not appear to affect adult HPA function, whereas ELS from age 6 to11 years results in exaggerated adult cortisol response to stress, and ELS from age 12 to 16 years is associated with blunting (54). These differing outcomes might be due to age-dependent variance in neural plasticity (42) and the impact of puberty (55). In any case, adults who have experienced ELS all appear to have smaller hippocampal volumes (56, 57), regardless of age of trauma and differing effects on HPA function. Stress in adulthood is also a risk factor for developing MDD, with up to 75% of MDD episodes following stress exposure (58, 59).

#### Gender

Female gender is an established risk factor for MDD (60), which has generated interest into probing whether females show higher magnitude cortisol responses to stress than males. A simple relationship, however, has not been established. When exposed to a stressful public speaking task, females in luteal phase showed similarly elevated salivary cortisol levels to men, whereas women in follicular phase, or on oral contraceptives, actually had lower cortisol levels than men and luteal phase women (61). The total serum cortisol level did not differ between groups (61). In contrast, higher cortisol levels following dexamethasone challenge were found in females, but not males, in a depressed cohort (62). In this study, higher cortisol was associated with greater burden of neurovegetative symptoms, providing additional evidence that cortisol abnormalities appear to be positively correlated with depressive severity (62). However, the findings that males with MDD can have higher baseline cortisol levels than females with MDD (63, 64) argue against there being a simple relationship between overall cortisol level and the increased incidence of MDD in women.

A clearer picture emerges from the finding that males with MDD and matched male controls show similar baseline cortisol levels, whereas females with MDD have significantly higher baseline cortisol than their matched controls (65). This suggests that the increased incidence of MDD in females might be better explained by intra-gender cortisol level variability (65). Women show differences in HPA regulation based on menopausal status, with menopausal depressed women being more likely to be corticotrophin non-suppressors than pre-menopausal women (66). Women in the luteal phase also show decreased cortisol suppression to DST compared to results obtained in the follicular phase (60). This may be due to a decrease in GR density during the luteal phase changing negative feedback sensitivity to dexamethasone, resulting in phase dependent intra-individual cortisol response variability (60). Young et al. hypothesize that it is this variable, rather than simply higher, female HPA responsiveness to stress across menstrual phase, and then across the lifetime, which explains the greater incidence of MDD in women (60). In a similar manner to the relationship between stress tasks, cortisol, and major depression, it again appears that the variability, rather than magnitude, of the cortisol response that correlates with MDD.

### Relationship Between Cortisol and MDD Treatment

#### Antidepressants

For monoamine reuptake inhibitor antidepressants, acute dosing is consistently associated with an increase in serum cortisol levels (see **Supplementary Table 1**). For example, in healthy volunteers, single infusions of the Selective serotonin reuptake inhibitor (SSRI) citalopram (20 mg), fluvoxamine (100 mg), and fluoxetine (80 mg) all produced increases in serum cortisol (67–69). Likewise, acute administration of reboxetine (4 mg), a noradrenaline reuptake inhibitor (NRI), and venlafaxine, a serotonin and noradrenaline reuptake inhibitor antidepressant (SNRI), also increased the level of cortisol in healthy subjects (70, 71). The ability of various monoamine reuptake inhibitor antidepressants to rapidly increase cortisol levels after acute administration might be due to increased monoamine concentrations directly affecting anterior pituitary hormone secretion (72).

In contrast to the monoamine reuptake inhibitors, a single dose of mirtazapine (15 mg) decreased serum cortisol, ACTH, and urinary free cortisol in healthy male volunteers (73). Mirtazapine is a presynaptic alpha2 and postsynaptic serotonin 5-HT_2_, 5-HT_3_ receptors and histamine H_1_ receptor antagonist that does not use reuptake inhibition to increase serotonin and noradrenaline levels (74). Modulation of these receptors in the rodent hypothalamus can inhibit CRH release, which may explain mirtazapine's ability to rapidly decrease cortisol levels in humans (8).

In studies of MDD patients, chronic antidepressant treatment has also been shown to normalize cortisol levels, irrespective of their differing pharmacological profiles (see **Supplementary Table 1**) (8). It has been suggested that normalization of GR function is the common antidepressant mechanism that allows improved negative feedback to the HPA and restoration of normal neuroendocrine function (75). For example, in a study where MDD and controls had similar baseline urinary cortisol levels, the clinical response after treatment with fluoxetine over 4 months was associated with a reduction in urinary cortisol (76).

Reductions in cortisol level are not, however, consistently associated with the clinical response to antidepressants (see **Supplementary Table 1**). For example, in a comparative study of 94 MDD patients treated with either amitriptyline (a tricyclic antidepressant; 150 mg) or paroxetine (a SSRI antidepressant; 40 mg), only the amitriptyline responders showed a decrease in salivary cortisol over 5 weeks, despite the similar clinical efficacy of these drugs (77). The consistency of the antidepressant effect on cortisol can also vary over time (see **Supplementary Table 1**). For example, after 7 days of mirtazapine treatment, the serum cortisol level decreased in 12 MDD responders for 5 weeks but then increased to higher levels than baseline, and was ultimately unable to distinguish them from non-responders (78).

#### Antipsychotics and Lithium

There is increasing evidence that atypical antipsychotics are efficacious in the treatment of MDD (8). The older generation “typical” antipsychotics was mostly limited to antagonizing dopaminergic D_2_ receptors, whereas newer “atypical” antipsychotics have additional antagonist activity at 5-HT_2A_, potentially allowing direct modulation of the hypothalamus to influence cortisol levels (8). For example, in healthy controls, acute dosing with the atypical antipsychotics olanzapine, quetiapine, and ziprasidone has been reported to reduce cortisol levels, whereas the typical antipsychotic haloperidol had no effect (79, 80). With respect to chronic dosing, after 1 week of treatment with quetiapine (300 mg), MDD patients had a significant reduction in cortisol in response to dexamethasone challenge; however, after 5 weeks cortisol secretion had increased to near pre-treatment levels (81). Lithium treatment in mood disorders has also been associated with increased cortisol levels in response to dexamethasone and DST challenge without reliably mapping to clinical response (82, 83).

#### GR Antagonists and Cortisol Synthesis Inhibitors

Probing the relationship between cortisol and mood disorder, the GR antagonist mifepristone has gathered considerable interest as a potential treatment for MDD. While initially showing promise for the treatment of psychotic depression (35, 84), further work has shown that its ameliorative effect is limited only to psychotic, rather than depressive, symptoms (85, 86). This result may be due to the wide variation in serum mifepristone concentrations across patients despite standard dosing (87), with higher serum concentrations associated with reducing psychotic symptoms only (34). Somewhat paradoxically, mifepristone, which appears to increase cortisol levels (85), reached Phase 3 trials, despite elevated cortisol being associated with worsening psychosis *via* increased dopaminergic tone (33). Mifepristone's modest antipsychotic mechanism remains poorly understood and interest in further antidepressant trials has waned after three failed Phase 3 studies (88).

Agents that block cortisol synthesis, rather than acting directly at the GR, have also been pursued. Metyrapone is an aromatic ketone that inhibits the synthesis of cortisol and has long been of interest for the treatment of MDD (89). More recent trials have confirmed that metyrapone is associated with an improvement in mood in MDD patients (90, 91) but beneficial effects do not appear to be related to cortisol levels and its underlying mechanism(s) remain unclear (92). Another cortisol synthesis inhibitor, ketoconazole, has only produced inconclusive results in MDD (93, 94). Notably, several HPA modulating agents showing promise in preclinical studies remain in the early stages of investigation and are yet to reach Phase 3 clinical trials (88, 95).

#### Electroconvulsive Therapy

Electroconvulsive therapy (ECT) is a somatic treatment for MDD that has been shown to be particularly efficacious in psychotic and melancholic depression (96). In a study of nine patients with psychotic MDD, ECT produced a reduction in cerebrospinal fluid CRH (97). Thirty minutes after treatment, ECT produced an increase in serum cortisol in 13 patients with melancholic MDD (98). In two studies of unspecified MDD, ECT led to a normalization of serum cortisol with resolution of depressive symptoms (99, 100).

#### Confounders to Interpreting Cortisol in MDD

Cortisol response to stress can be measured across three phases: (a) basal, or unstressed, phase; (b) reactivity phase, where cortisol rises in response to a stressor; and (c) recovery phase; where cortisol returns to baseline following stress. These phases are regulated by different receptors, with GR regulating cortisol during reactivity phase, and MR regulating cortisol at periods of baseline activity. In a meta-analysis, Burke et al. reviewed these varying cortisol phases in depressed patients versus controls (101). Depressed patients tended to show a relatively flat unresponsive pattern of cortisol secretion with both blunted stress reactivity and impaired recovery phases. This association also appears to hold true for prospective MDD risk. In patients who had undergone coronary artery bypass grafting (a stressor), those whose salivary cortisol returned to normal more slowly during the recovery phase were most likely to have MDD a year later (102). Similarly, in adolescents with a history of stressful life events, those who also had a history of recurrent depression also had a persistently elevated reactivity phase to stress (32). The meta-analysis showed that blunted response curves are most strongly associated with severe MDD. In contrast, non-depressed controls showed greater reactivity variability and faster recovery phase following stressors. Conclusions from the meta-analysis were limited, however, as it included studies focusing on total salivary, rather than free, cortisol fraction.

Salivary cortisol has frequently been used to assay stress in MDD studies. While being relatively inexpensive and pain-free to test in subjects, it is limited by only being able to measure unbound free cortisol (103). In contrast, serum cortisol assays are able to measure unbound and protein bound cortisol fractions but have the disadvantage of being more difficult to collect and the added confounder of stress associated with venepuncture (104). There is a high correlation between salivary cortisol and free serum cortisol levels, which is maintained during DST and ACTH challenge (105). The usefulness of this correlation, however, relies on the presumption that only free serum cortisol is biologically active (103). This notion has been challenged by the finding that cortisol bound to cortisol binding globulin (CBG) can have physiological effects on tissue (106). Furthermore, the distinction between free and bound cortisol is not very clear, with hepatic uptake of bound cortisol being threefold faster than free cortisol followed by rapid dissociation, suggesting that CBG may be acting as an active delivery mechanism, rather than passive storage, in serum (106). In contrast to its relationship with free serum cortisol, salivary cortisol does not have a linear relationship with total cortisol. Instead, the ratio of salivary cortisol to total cortisol is concentration dependent, with 1–2% at lower concentrations and rising to 8–9% at higher concentrations (105). Salivary cortisol levels also rise dramatically once CBG is saturated *via* sex steroids such as during the follicular phase, pregnancy, and usage of the oral contraceptive pill (61, 106–107). Confounders such as these have likely contributed to the marked heterogeneity of results in a meta-analysis of salivary cortisol levels in 1,354 MDD patients and 1,052 controls, which concluded that salivary cortisol alone was unable to distinguish MDD (108). Recent work has also shown how differences in dexamethasone metabolism, rather than MDD symptoms, can better explain variance in cortisol levels (109), though studies have attempted to control for this by directly sampling serum dexamethasone (14). To counter difficulties with salivary cortisol, it is possible to measure free and bound fractions of serum cortisol directly *via* liquid chromatography-tandem mass spectrometry; however, reference ranges using this technique for suppression tests are yet to be established (110). Furthermore, the DST only probes GR, whereas the less frequently used, but more physiologically relevant, prednisolone suppression test examines both GR and MR function (111), both of which interact abnormally in MDD pathophysiology.

## Understanding the Neurobiological Mechanisms of Depression: Modelling MDD in Preclinical Studies

Despite progress in the development of non-invasive techniques to study MDD in humans, detailed examination of the molecular and cellular mechanisms and neural circuitry underpinning the disorder remains limited. Some limitations can be addressed *via* animal models that critically confer the ability to study cause and effect relationships under controlled conditions. For example, although human genome studies have revealed genes associated with depression (112), additional human studies cannot prospectively address how such genes might cause depression. In contrast, being able to manipulate these genes in transgenic animals provides an opportunity to correlate depression-associated behaviors with candidate mechanisms (113).

Different behavioral responses are scored and quantified in preclinical models as surrogates of mood-related changes. For example, in rodent models, the amount of time spent immobile in the forced swim test and the tail suspension test are used to measure behavioral despair, which is thought to be analogous to the hopelessness seen in MDD (114). The sucrose preference test has been used to determine anhedonic behavior in rodents, potentially corresponding to the diminished interest in pleasurable experiences seen in depressed patients (114). In rodent colonies, the social interaction test has been used to model the social withdrawal seen in MDD (115). Due to the high comorbidity of depression and anxiety (116), behavioral tests that examine rodent anxiety, such as the elevated plus maze, novelty-suppressed feeding, open field, and dark-light box tests, are also used to study depression (117, 118). Utilizing multiple behavioral assays thus provides an experimental system that features analogues of human MDD and is open to detailed experimental manipulation.

Given that both susceptible genes and environmental risk factors are implicated in the development of depression, most animal models employ a combination of genetic manipulation and environmental stressors to produce animals that exhibit depression-like behaviors. The evolutionary conservation of the stress response brain circuitry between humans and rodents remains fundamental to translational studies (**Figure 1**). In rodents, stress induces secretion of corticosterone *via* the HPA axis, with rodent corticosterone and human cortisol having functionally equivalent roles (119).

## Corticosterone Levels in Genetic Models of Depression

Most genetic models of MDD in rodents are generated *via* selective breeding. Animals with the desired features are bred over several generations, yielding inbred strains that reliably exhibit specific physiological or behavioral abnormalities (120). For example, Flinders sensitive line (FSL) rats display both depression-like behavior (121) and vulnerability to stress-induced anhedonia-like behavior (122). These behavioral traits are also associated with elevated corticosterone levels (123–125). Wistar-Kyoto (WKY) rats are characterized by depression-like and anxiety-like behaviors (126), and exhibit an upregulated basal serum corticosterone level at the start of the dark cycle, which is reminiscent of the circadian rhythm abnormalities seen in human MDD (127, 128). Importantly, prolonged corticosterone stress responses and DST non-suppressibility have been observed in WKY rats (128, 129). Congenitally learned helpless (cLH) rats show pronounced helplessness behavior without prior exposure to uncontrollable shock (130). Baseline corticosterone levels are similar between cLH rats and controls; however, cLH rats experiencing acute stress have a decreased cortisol response compared with adult controls (131, 132), reminiscent of the differences in the stress cortisol response between traumatized and non-traumatized MDD patients.

## Corticosterone Levels in Environmental Models of Depression

Chronic mild stress (CMS) paradigms are often used to model MDD in rodents due to their ability to induce behavioral despair, and anxiety-like and anhedonia-like behaviors (133). In CMS models, there is a consistent increase in serum corticosterone following stressor exposure (134–138). Notably, reducing corticosterone levels *via* pharmacological inhibition of corticosterone synthesis or adrenalectomy has been shown to block the development of depressive behaviors in CMS models (139, 140). Compared to physical stressors, such as restraint, damp bedding, and food deprivation, social stressors, such as social isolation (141) and social defeat (142), tend to more reliably produce depression-like behaviors but are associated with variable basal corticosterone levels while leading to increase in corticosterone levels in response to acute stress (141–150).

Models of ELS, induced by maternal separation or limited nesting, have also been used to recapitulate the association between adverse childhood events and MDD (151, 152). In rodents, the effects of ELS on corticosterone levels are highly variable, being stressor-specific as well as dependent on the developmental period during which the stressors are administered and the age at which animals are assessed (152–159). This is reminiscent of the weak association between early life trauma and dysregulated HPA response to acute stress in adults.

Collectively, the findings from rodent models display some similarities to those observed in humans, where it is the variable responsiveness to repeated stress, rather than the basal level of corticosterone, which is associated with depression-like behavior.

## Modelling Depression Using Exogeneous Corticosterone Administration

To simulate the HPA dysregulation seen in the genetic and stress-based models of depression, researchers have used exogeneous corticosterone administration in rodents (160). Robust and highly reproducible anxiety- and depression-like behaviors have been reported following chronic oral administration of corticosterone, which may or may not lead to an elevation in the level of serum corticosterone (133, 161–169). In addition to the behavioral changes, chronic exogeneous corticosterone is associated with several neurobiological changes seen in MDD models including the disruption of adult hippocampal neurogenesis (170), and decreased hippocampal brain derived neurotrophic factor (BDNF) (171). Chronic treatment with classical antidepressants such as fluoxetine and imipramine (160, 172–174), as well as a single dose of faster-acting antidepressant ketamine (164, 175), have been shown to reverse corticosterone-induced depression-like behaviors. In a subset of these studies, beneficial behavioral effects of antidepressant treatment was accompanied by normalization of serum corticosterone levels (172–174). Notably, sub-chronic treatment with GR selective antagonist, RU-43044, has also been shown to reverse corticosterone-induced depression-like behavior (176) suggesting GR as a potential molecular target to combat HPA axis dysregulation.

## Classical Antidepressants and Corticosterone

Mirroring findings in humans, acute and sub-chronic treatment with classical antidepressants have been reported to increase corticosterone levels in rodents (see **Supplementary Table 2**). For example, in non-stressed animals, a single injection of fluoxetine (10 mg/kg) or imipramine (30 mg/kg) increased serum corticosterone levels (177, 178). It has been suggested that following antidepressant administration, hippocampal GR and mineralocorticoid (MR) receptors are downregulated, which reduces HPA negative feedback, thereby leading to an increase in corticosterone (179). This hypothesis is supported by the finding that 9 days of fluoxetine (10 mg/kg) and venlafaxine (10 mg/kg) treatment results in downregulation of hippocampal GRs and MRs (180). In contrast to these effects, chronic antidepressant treatment had no effect on basal corticosterone levels in non-stressed rodents. For example, 24 days of either paroxetine, (7.5 mg/kg) (181), venlafaxine (10 mg/kg) (181), or desipramine (7.5 mg/kg) (181, 182) did not affect basal serum corticosterone levels.

Paralleling findings in human MDD described above (summarized in **Supplementary Table 1**), multiple antidepressants have been shown to normalize corticosterone levels in stressed animals (see **Supplementary Table 2**). For example, treatment with fluoxetine for more than 2 weeks normalized basal corticosterone levels in chronic unpredictable stress and social defeat stress rodent models (183–185). Similarly, chronic treatment with venlafaxine or imipramine normalized basal corticosterone in chronic unpredictable stress and prenatal stress exposed rodents (186–190).

## Atypical Antidepressants, Lithium, and Corticosterone

In rodents, the effect of atypical antidepressants on corticosterone levels is similar to those seen with classical antidepressants (see **Supplementary Table 2**). For example, a single injection of mirtazapine increased basal corticosterone levels (177), whereas a longer treatment period had no effect on either basal or post-stress levels (177, 191). In chronic unpredictable stress and prenatal stress, chronic mirtazapine treatment normalized basal and post-stress corticosterone (188, 190). Chronic treatment with tianeptine (10 mg/kg), a tricyclic antidepressant that locks the serotonin transporter into the open position, also normalized basal corticosterone in a prenatal stress model (190). Single (1.4 meq Li^+^/kg) and repeated (45 meq Li^+^ mixed with 1 kg food, 6 days) lithium treatment of non-stressed rats increased corticosterone levels (192). However, in socially isolated mice, a single high dose (10 mg/kg) injection of lithium decreased post-stress, but not basal, corticosterone levels (193). Similarly, repeated lithium treatment at a moderate dose (2.5 mg/kg) decreased basal corticosterone levels in a chronic unpredictable stress model in rats (194).

## Conclusions and Future Perspectives

In summary, clinical studies show that MDD is most consistently associated with stress-induced variability in HPA axis response rather than absolute levels of cortisol. Elevated baseline cortisol levels are only seen in more severe subtypes of MDD and are conspicuously absent in those with chronic or atypical presentations. Furthermore, a reduction in cortisol is not reliably associated with successful treatment with monoaminergic antidepressants, regardless of type. Accordingly, baseline cortisol, or corticosterone levels, cannot be used as a simple proxy for modelling MDD in general, or evaluating treatment response in either humans or animals.

Recent work has resulted in a rapid expansion of the potential mechanisms that could underlie the increasingly complex relationship between HPA axis dysfunction and MDD. Arginine vasopressin (AVP), a peptide hormone secreted from the posterior pituitary, acts synergistically with CRH to increase the release of ACTH and cortisol (**Figure 1**) (196). AVP is increased in MDD (195, 196), possibly contributing to the variable cortisol response to stress challenge in these populations. Interestingly, recent work has also shown that melancholic MDD is specifically associated increased AVP activity (197), which is linked to increased vasopressin V3 receptor expression (198). It remains unknown whether such changes are also present in milder forms of MDD. MDD also features HPA axis changes at the receptor level. GR and MR act in a complementary fashion during the stress response but are thought to be in imbalance in MDD (10, 199), which may lead to variable cortisol expression during stress *via* competition between these pathways. In addition to this abnormal relationship between MR and GR, negative feedback through GR is unpredictably impaired in MDD, further exacerbating cortisol variability (200, 201). In addition to these emerging receptor and endocrine mechanisms, genetics and epigenetic are also likely contributing to the varied results in extant literature.

Evidence from clinical and animal studies suggests that genetic and epigenetic mechanisms link chronic stress, HPA axis dysregulation, and alterations to glucocorticoid levels and signaling. For example, polymorphisms in promoter region of the GR gene, *NR3C1*, have been associated with either increased sensitivity (202, 203) or resistance to cortisol (204). There is also increased DNA methylation of hippocampal *NRC31* associated with depression, including in the brains of suicide victims with a history of ELS (205, 206). Expression of GR mRNA is decreased in those with a history of childhood sexual abuse (205) and GR gene methylation is increased in children who have experienced trauma (207). In addition to genetic and epigenetic regulation of the GR gene, polymorphism in FK506 binding protein (*FKBP5*), an important regulator of GR function, has been associated with DST hyperresponsiveness (208). The importance of this gene to stress-related disorders has been further supported by studies of *FKBP5* knockout mice that show an increased sensitivity of GRs (209, 210). Similarly, polymorphism in the CRH receptor (*CRHR1*) gene has also been linked to MDD (211) and is associated with HPA axis dysregulation.

The finding that a diverse range of antidepressants rapidly stabilize cortisol levels following acute administration could potentially explain why their beneficial effects are observed within hours of commencement compared to placebo (212). We speculate that antidepressants might be overriding ELS-induced adult cortisol variability in response to stress, which allows recovery to commence. However, at present, the inconsistent relationship between chronic antidepressant administration, clinical response, and cortisol remains difficult for any mechanistic interrogation. Fortunately, the data showing that multiple antidepressants have similar effects on stress hormones in humans and rodents increases confidence that there is sufficient evolutionary conservation between species to carefully model HPA axis dysregulation in rodents to further probe MDD neurobiology. While rodent models have proved extremely valuable in gaining mechanistic insights into the pathophysiology, emerging studies using the non-human primates have begun to offer additional dimensions to the endocrine and behavioral correlates of chronic stress induced MDD (213, 214). Exploring the effects of HPA axis modulation in these animal models could provide a bridge to improve our mechanistic understanding of how changes in cortisol cascade to abnormalities in metabolism, gene regulation, and immune function, all of which have been independently implicated in MDD. This combined with high-throughput longitudinal monitoring of group-housed animals, as reported recently for mice (215), could yield a variety of behavioral readouts of individual animals and help stratify vulnerable versus resilient subpopulations in response to various stress such as early life or social stress.

An ideal rodent or non-human primate model should therefore reliably produce exaggerated or blunted HPA axis responses to acute or chronic stress *a priori*, which may, or may not, alter baseline cortisol levels. Such a model would provide an evidence-based neurochemical environment that can inform subsequent behaviors as targets for reversal. The value of such a model would be to correlate the established biomarker of HPA dysregulation with multiple emerging biomarkers using modern neuroscience tools. For example, recent advances in small animal imaging using magnetic resonance imaging (MRI) confers the ability to directly measure structural and functional changes in the rodent brain, thereby providing a bridge to translate findings from animals to humans. Although structural MRI has been used to report robust subcortical brain alterations in MDD (216), a recent approach (217) exploiting differences in the resting state connectivity between brain regions among MDD patients to define novel subtypes of depression has excited clinical and preclinical researchers alike. We therefore envisage that structural and functional MRI-based changes, together with HPA tissue biomarkers, will correlate with clinical subtypes of MDD and guide new treatment approaches.

Recent advances in identifying genetic risks (112), together with access to brain-wide transcriptome data from post-mortem clinical samples (218), will also advance our understanding of brain region-specific roles for select genes and molecular networks in MDD. Defining the roles of these genes, as well as selective manipulation of the underlying brain circuits, using tools such as optogenetics, in HPA informed animal models will likely drive further insight into MDD.

Thus, a research pathway that encompasses multi-modal investigations, including molecular, cellular, neurocircuitry, and behavioral studies in preclinical models, and which is informed and refined by clinical findings will accelerate understanding of MDD and provide multiple strategies to effectively diagnose and treat this heterogeneous disorder. It is vital that we persevere and strengthen such interactions between preclinical and clinical research to drive multiple iterations of this pathway.

## Author Contributions

LSN and DJ conceptualized the review. LSN, MB, MZ, and DJ conducted critical evaluation of literature and contributed to the draft manuscript. LSN and DJ wrote the paper.

## Conflict of Interest

The authors declare that the research was conducted in the absence of any commercial or financial relationships that could be construed as a potential conflict of interest
