# Supplementary material for: Cortisol and Major Depressive Disorder—Translating Findings From Humans to Animal Models and Back
Source: Front Psychiatry. 2020 Jan 22;10:974. doi: 10.3389/fpsyt.2019.00974 (PMC6987444; doi:10.3389/fpsyt.2019.00974)
Supplement: Supplementary file 1 [file Table_1.docx]

**Supplementary Table 1**: Cortisol levels in humans following psychotropic treatment.

| **Anti-depressant class** | **Reference** | **Treatment** | **Duration** | **Subjects** | **Depression subtype** | **Method of sampling** | **Effects on cortisol** |
| --- | --- | --- | --- | --- | --- | --- | --- |
| **AP** | Cohrs et al., 2006 (1) | Olanzapine 5mg,  Quetiapine 50mg or  Haloperidol 3mg | 4 days | 11 males | Healthy subjects | Plasma, urine | Olanzapine ↓  Quetiapine ↓  Haloperidol — |
|  | Meier et al., 2005 (2) | Ziprasidone 40mg | Acute dosing | 11 males | Healthy subjects | Urine | ↓ |
| **Atypical AD** | Rao et al., 2005 (3) | Bupropion 150mg | 8 weeks | 10 females, 10 males | Unipolar major depression | Urine | — |
|  | Schüle, Baghai, Eser, Häfner, et al., 2009 (4) | Reboxetine 8mg or mirazapine 45mg | 5 weeks | 61 females, 53 males | Melancholic major depression | Plasma + DEX/CRH | Reboxetine —  Mirtazapine ↓ |
|  | Schüle et al., 2004 (5) | Reboxetine 4mg | Acute dosing | 12 males | Healthy subjects | Plasma | ↑ |
|  | Schüle et al., 2006 (6) | Reboxetine 8mg or mirtazapine 45mg | 5 weeks | 23 females, 17 males | Unipolar major depression | Plasma + DEX/CRH | Reboxetine ↓  Mirtazapine ↓ |
|  | Tse and Bond, 2005 (7) | Reboxetine 4mg | Acute dosing | 23 females, 17 males | Healthy subjects | Saliva | ↑ |
|  | Hill et al., 2003 (8) | Reboxetine 4mg | Acute dosing | 14 females, 10 males | Healthy subjects | Plasma and saliva | ⭡ |
|  | Schmid et al., 2006 (9) | Mirtazapine 15-45mg | 28 days | 7 females,  3 males | Unspecified | Plasma + DEX/CRH | ↓ |
|  | Schüle, Baghai, Eser, Schwarz, et al., 2009 (10) | Mirtazapine 45mg | 35 days | 19 females, 4 males | Melancholic | Plasma | ↓ |
|  | Laakmann et al., 2004 (11) | Mirtazapine 45mg | 3 weeks | 8 females,  4 males | MDD | Saliva | ↓ |
|  | Schule et al., 2003 (12) | Mirtazapine 45mg | 3 weeks | 14 females, 6 males | MDD | Urine | ↓ |
|  | Schüle et al., 2003 (13) | Mirtazapine 45mg | 1 week | 23 females, 17 males | MDD | Plasma + DEX/CRH | ↓ |
|  | Schüle et al., 2002 (14) | Mirtazapine 15mg | Acute dosing | 12 males | Healthy subjects | Plasma, urine | ↓ |
| **ECT** | Deakin et al., 1983 (15) | ECT twice weekly | 4 weeks | 62 subjects | Endogenous depression | Plasma | ↑ |
|  | Zis et al., 1996 (16) | Unilateral ECT three times per week | 1 week | 7 females,  3 males | MDD | Plasma | ↑ |
|  | Yuuki et al., 2005 (17) | Bifrontal ECT and usual antidepressant | After completing acute ECT course | 3 females,  4 males | Unipolar and bipolar major depression | Plasma + DEX/CRH | ↓ |
|  | Burgese and Bassitt, 2015 (18) | Bilateral ECT | 7 ECT sesions | 7 females, 4 males | MDD | Plasma | ↓ |
| **MAO** | Kin et al., 1997 (19) | Moclobemide 400mg | 7 weeks | 95 subjects | MDD | Plasma + DEX/CRH | — |
| **Mood Stabiliser** | Bschor et al., 2011 (20) | Lithium 900mg | 28 days | 15 females, 15 males | MDD | Plasma + DEX/CRH | ↑ |
|  | Bschor et al., 2003 (21) | Lithium titrated to levels 0.5-1.0 mmol/L | 3-4 weeks | 12 females, 13 males | Unipolar major depression | Plasma – DEX | ↑ |
|  | Bschor et al., 2002 (22) | Lithium titrated to levels 0.5-1.0 mmol/L | 4 weeks | 17 females, 13 males | Unipolar major depression | Plasma + DEX/CRH | ↑ |
| **Multiple** | B.S. et al., 1993 ^3^ | Phenelzine 45mg, imipramine 150mg, nortriptyline 75mg fluoxetine 20mg or ECT | Until discharge | 22 females, 8 males | Unipolar major depression | Plasma + DEX/CRH | ECT + medication ↓ |
|  | Horstmann et al., 2009 (24) | SSRIs, Mirtazapine, venlafaxine, TCA | Acute  dosing | 88 females, 72 male | Unspecified, unipolar and bipolar depression | Plasma + DEX/CRH | SSRIs, Mirtazapine, venlafaxine, TCA |
| **Other** | Amsterdam et al., 1989 (25) | Clonidine 2.5 μg/kg | Acute dosing | 18 subjects | 12 with melancholic features, 6 unspecified | Plasma | ↓ |
| **SNRI** | Ninan et al., 2014 (26) | Desvenlafaxine 50mg | 84 days | 188 females, 97 males | MDD without psychotic features | Saliva | — |
|  | Wang et al., 2012 (27) | Duloxetine 60-120mg | 12 weeks | 15 females, 11 males | MDD | Serum | — |
|  | Daffner-Bugía et al., 1996 (28) | Venlafaxine up to 75mg | Acute dosing | 6 males | Healthy subjects | Plasma | ↑ |
| **SNRI/Atypical AD** | Scharnholz et al., 2010 (29) | Mirtazapine up to 60mg or venlafaxine up to 225mg | 28 days | 65 females, 30 males | Unspecified | Saliva | Mirtazapine ↓, Venlafaxine — |
| **SSRI** | Papakostas et al., 2000 (30) | Citalopram 20mg prior to ECT | Acute dosing | 10 females | Unipolar major depression | Plasma | — |
|  | Hinkelmann et al., 2012 (31) | Escitalopram up to 20mg | 21 days | 37 females, 15 males | Unspecified | Saliva | ↓ |
|  | Thakore et al., 1997 (32) | Sertraline 50mg or paroxetine 20mg | After efficacy | 7 females | Melancholic | Plasma | ↓ |
|  | Piwowarska et al., 2012 (33) | Fluoxetine 20mg | 56 days | 14 females, 7 males | Unspecified MDD | Plasma | — |
|  | Kauffman et al., 2005 (34) | Citalopram up to 40mg | 56 days | 14 females | Unspecified MDD | Serum | — |
|  | Vythilingam et al., 2004 (35) | Fluoxetine up to 40mg | 6 ± 2 months | 23 females, 15 males | 11 melancholic, 6 atypical | Urinary 24 hour cortisol, plasma DEX/CRH | ↓ |
|  | Bschor et al., 2012 (36) | Citalopram up to 40mg | 28 days | 24 females, 6 males | MDD | Plasma – DEX/CRH | ↑ |
|  | Nikisch et al., 2005 (37) | Citalopram 40mg | 16 weeks | 11 females, 9 males | MDD | Plasma – Dex/CRH, CSF | ↓ |
|  | Zobel et al., 2004 (38) | Citalopram up to 40mg | 36 days | 27 females, 38 males | Unipolar major depression without psychotic features | Plasma – DEX/CRH | ↓ |
|  | Ruhé et al., 2015 (39) | Paroxtine up to 50mg | 12 weeks | 46 females, 24 males | Non-psychotic MDD | Saliva | ↓ |
|  | Šagud et al., 2002 (40) | Sertraline 50mg | 24 weeks | 15 females | MDD | Plasma | ↑ |
|  | Jazayeri et al., 2010 (41) | Fluoxetine 20mg | 8 weeks | 10 females, 4 males | Non-psychotic MDD | Plasma | ↓ |
|  | Seifritz et al., 1996 (42) | Citalopram 20mg | Acute dosing | 8 males | Healthy subjects | Plasma | ↑ |
|  | Demisch et al., 1986 (43) | Fluvoxamine 150mg | Acute dosing | 6 males | Healthy subjects | Plasma | ↑ |
| **SSRI/AP** | Sarubin et al., 2014 (44) | Quetiapine XR 300mg or escitalopram 10mg | 35 days | 60 subjects | Unspecified | Plasma + DEX/CRH | Quetiapine ↓  Escitalopram ↑ |
|  | Nothdurfter et al., 2014 (45) | Escitalopram up to 20mg +/- quetiapine 200mg | 35 days | 19 females, 21 males | MDD | Plasma + DEX/CRH | Escitalopram —  Escitalopram + quetiapine — |
| **SSRI/Atypical AD** | Straneva-Meuse et al., 2004 (46) | Paroxetine up to 50mg or bupropion up to 450mg | 8 weeks | 34 subjects | MDD | Plasma | Paroxetine ↓  Bupropion ↓ |
| **SSRI/TCA** | Nickel et al., 2003 (47) | Paroxetine up to 40mg, tianeptine up to 75mg | 42 days | 23 females, 21 males | MDD | Plasma + DEX/CRH | Paroxetine ↓ Tianeptine ↓ |
|  | Deuschle et al., 2003 (48) | Paroxetine up to 40mg and amitriptyline up to 150mg | 5 weeks | 85 females, 42 males | Unipolar MDD | Saliva | Amitriptyline ↓  Paroxetine — |
|  | Mück-Šeler et al., 2002 (49) | Paroxetine 20mg or tianeptine 37.5mg | 4 weeks | 36 females | Unipolar MDD | Plasma | Paroxetine —  Tianeptine — |
|  | Weber-Hamann et al., 2007 (50) | Paroxetine 40mg or amitriptyline 150mg | 5 weeks | 25 females, 12 males | MDD | Saliva | Paroxetine —  Amitriptyline ↓ |
|  | Manthey et al., 2011 (51) | 309 SSRI users, 49 TCA users, 100 other antidepressant users and 1,068 medication free subjects | 2 days | 1,526 subjects | Current or past diagnosis of a depressive and/or anxiety disorder | Saliva + DEX | TCAs ↓  SSRIs ↑ |
|  | Rota et al., 2005 (52) | Amitriptyline 150mg or fluvoxamine 200mg | 42 days | 22 females, 16 males | Unipolar MDD | Plasma | Amitriptyline —  Fluvoxamine — |
|  | Kopf et al., 2004 (53) | Amitriptyline 150mg or paroxetine 40mg | 35 days | 54 females, 24 males | MDD | Saliva | Amitriptyline ↓  Paroxetine — |
|  | Weber-Hamann et al., 2009 (54) | Amitriptyline 150mg or paroxetine 40mg | 5 weeks | 55 females, 22 males | Unipolar major depression | Saliva | Amitriptyline ↓  Paroxetine — |
|  | Skene et al., 1994 (55) | Fluvoxamine 100mg or desipramine 100mg | Acute dosing | 8 males | Healthy subjects | Plasma | Fluvoxamine ↑  Desipramine ↑ |
| **TCA** | Sachar et al., 1973 (56) | Imipramine | 7 days | Six subjects | Psychotic | Plasma | ↓ |
|  | Kin et al., 1997 (19) | Nortriptyline 75mg | 7 weeks | 95 subjects | MDD | Plasma + DEX | — |
|  | Heuser et al., 1996 (57) | Amitriptyline 75mg | 6 weeks | 32 females, 9 males | MDD | Plasma + DEX/CRH | ↓ |
|  | Carson and Halbreich, 1987 (58) | Amitriptyline 100-200mg | 4 weeks | 9 females and 6 males | Endogenous Major depression | Plasma + DEX | — |
|  | Schmider et al., 1995 (59) | Amitriptyline 75mg | 6 weeks | 31 females, 7 males | MDD | Plasma + DEX/CRH | ↓ |
|  | O’Keane et al., 1992 (60) | Amitriptyline up to 250mg, fluoxetine 20mg or ECT | 4 weeks | 11 females 10 males | MDD | Serum - D-fenfluramine | Amitriptyline —  Fluoxetine —  ECT — |
|  | Lisansky et al., 1987 (61) | Amitriptyline 150mg | 4 weeks | 3 females,  5 males | Melancholic major depression | Plasma + DEX | ↓ |
|  | Piwowarska et al., 2009 (62) | Clomipramine up to 150mg | 8 weeks | 12 females, 5 males | Unipolar or bipolar depression | Plasma | ↓ |
|  | Shapira et al., 1992 (63) | Clomipramine up to 250mg | 4 weeks | 5 females, 4 males | Endogenous major depression | Serum - D-fenfluramine | — |
|  | Coote et al., 1998 (64) | Desipramine 200mg or ECT | 4 weeks | 13 females, 13 males | Melancholic major depression | Plasma + Clonidine challenge | ↓ |
|  | Sonntag et al., 1996 (65) | Trimipramine 200mg or imipramine 150mg | 4 weeks | 20 males | Unipolar major depression | Plasma | Trimipramine ↓  Imipramine — |
|  | Künzel et al., 2009 (66) | Trimipramine 400mg or amitriptyline 200mg and haloperidol 7.5mg | 6 weeks | 34 females, 23 males | Psychotic major depression | Plasma + DEX/CRH | Trimipramine ↓  Amitriptyline + haloperidol ↓ |
|  | Holsboer-Trachsler et al., 1991 (67) | Trimipramine 200mg | 6 weeks | 7 females,  7 males | MDD | Plasma + DEX/CRH | ↓ |
| **TCA/AP** | Laakmann et al., 1984 (68) | Desipramine 50mg, clomipramine 25mg, sulpride 100mg or diazepam 10mg | Acute dosing | 36 males | Healthy subjects | Plasma | Desipramine ↑  Clomimipramine ↑  Sulpride —  Diazepam — |
| **TCA/ECT** | Linkowski et al., 1987 (69) | Amitriptyline 125-250mg or ECT | 6-8 weeks | 11 males | Unipolar and bipolar depression | Plasma + DEX | ↓ |
| **TCA/MAO** | Georgotas et al., 1986 (70) | Nortriptyline (titrate to plasma level 50-170ng/ml) or phenelzine titrated to platelet MAO inhibition ≥70% | 7 weeks | 42 females 30 males | MDD | Plasma + DEX | Nortriptyline —  Phenelzine — |

^ECT, Electro-convulsive Therapy; MAO, monoamine oxidase inhibitor; TCA, tricylic antidepressant; SSRI, selective serotonin reuptake inhibitor; SNRI, selective serotonin and noradrenaline reuptake inhibitor; AP, antipsychotic; DEX, dexamethasone; CRH, corticotropin releasing hormone; — No effect; ↓^ ^Reduction; ↑ Increase^

**References**

1. Cohrs S, Röher C, Jordan W, Meier A, Huether G, Wuttke W, Rüther E, Rodenbeck A. The atypical antipsychotics olanzapine and quetiapine, but not haloperidol, reduce ACTH and cortisol secretion in healthy subjects. *Psychopharmacology (Berl)* (2006) **185**:11–18. doi:10.1007/s00213-005-0279-x

2. Meier A, Neumann AC, Jordan W, Huether G, Rodenbeck A, Rüther E, Cohrs S. Ziprasidone decreases cortisol excretion in healthy subjects. *Br J Clin Pharmacol* (2005) **60**:330–336. doi:10.1111/j.1365-2125.2005.02431.x

3. Rao U, Ott GE, Lin KM, Gertsik L, Poland RE. Effect of bupropion on nocturnal urinary free cortisol and its association with antidepressant response. *J Psychiatr Res* (2005) **39**:183–190. doi:10.1016/j.jpsychires.2004.01.009

4. Schüle C, Baghai TC, Eser D, Häfner S, Born C, Herrmann S, Rupprecht R. The combined dexamethasone/CRH test (DEX/CRH test) and prediction of acute treatment response in major depression. *PLoS One* (2009) **4**:e4324. doi:10.1371/journal.pone.0004324

5. Schüle C, Baghai T, Laakmann G. Mirtazapine Decreases Stimulatory Effects of Reboxetine on Cortisol, Adrenocorticotropin and Prolactin Secretion in Healthy Male Subjects. *Neuroendocrinology* (2004) **79**:54–62. doi:10.1159/000076046

6. Schüle C, Baghai TC, Eser D, Zwanzger P, Jordan M, Buechs R, Rupprecht R. Time course of hypothalamic-pituitary-adrenocortical axis activity during treatment with reboxetine and mirtazapine in depressed patients. *Psychopharmacology (Berl)* (2006) **186**:601–611. doi:10.1007/s00213-006-0382-7

7. Tse WS, Bond AJ. Sex differences in cortisol response to reboxetine. *J Psychopharmacol* (2005) **19**:46–50. doi:10.1177/0269881105048896

8. Hill SA, Taylor MJ, Harmer CJ, Cowen PJ. Acute reboxetine administration increases plasma and salivary cortisol. *J Psychopharmacol* (2003) **17**:273–275. doi:10.1177/02698811030173008

9. Schmid DA, Wichniak A, Uhr M, Ising M, Brunner H, Held K, Weikel JC, Sonntag A, Steiger A. Changes of sleep architecture, spectral composition of sleep EEG, the nocturnal secretion of cortisol, ACTH, GH, prolactin, melatonin, ghrelin, and leptin, and the DEX-CRH test in depressed patients during treatment with mirtazapine. *Neuropsychopharmacology* (2006) **31**:832–844. doi:10.1038/sj.npp.1300923

10. Schüle C, Baghai TC, Eser D, Schwarz M, Bondy B, Rupprecht R. Effects of mirtazapine on dehydroepiandrosterone-sulfate and cortisol plasma concentrations in depressed patients. *J Psychiatr Res* (2009) **43**:538–545. doi:10.1016/j.jpsychires.2008.07.003

11. Laakmann G, Hennig J, Baghai T, Schüle C. Mirtazapine acutely inhibits salivary cortisol concentrations in depressed patients. *Ann N Y Acad Sci* (2004) **1032**:279–282. doi:10.1196/annals.1314.038

12. Schule C, Baghai T, Rackwitz C, Laakmann G. Influence of mirtazapine on urinary free cortisol excretion in depressed patients. *Psychiatry Res* (2003) **120**:257–264. doi:10.1016/S0165-1781(03)00204-X

13. Schüle C, Baghai T, Zwanzger P, Ella R, Eser D, Padberg F, Möller HJ, Rupprecht R. Attenuation of hypothalamic-pituitary-adrenocortical hyperactivity in depressed patients by mirtazapine. *Psychopharmacology (Berl)* (2003) **166**:271–275. doi:10.1007/s00213-002-1356-z

14. Schüle C, Baghai T, Bidlingmaier M, Strasburger C, Laakmann G. Endocrinological effects of mirtazapine in healthy volunteers. *Prog Neuro-Psychopharmacology Biol Psychiatry* (2002) **26**:1253–1261. doi:10.1016/S0278-5846(02)00264-6

15. Deakin JFW, Ferrier IN, Crow TJ, Johnstone EC, Lawler P. Effects of ECT on pituitary hormone release: Relationship to seizure, clinical variables and outcome. *Br J Psychiatry* (1983) **143**:618–624. doi:10.1192/bjp.143.6.618

16. Zis AP, Yatham LN, Lam RW, Clark CM, Srisurapanont M, McGarvey K, Zis AP. Effect of stimulus intensity on prolactin and cortisol release induced by unilateral electroconvulsive therapy. *Neuropsychopharmacology* (1996) **15**:263–270. doi:10.1016/0893-133X(95)00206-S

17. Yuuki N, Ida I, Oshima A, Kumano H, Takahashi K, Fukuda M, Oriuchi N, Endo K, Matsuda H, Mikuni M. HPA axis normalization, estimated by DEX/CRH test, but less alteration on cerebral glucose metabolism in depressed patients receiving ECT after medication treatment failures. *Acta Psychiatr Scand* (2005) **112**:257–265. doi:10.1111/j.1600-0447.2005.00625.x

18. Burgese DF, Bassitt DP. Variation of plasma cortisol levels in patients with depression after treatment with bilateral electroconvulsive therapy. *Trends Psychiatry Psychother* (2015) **37**:27–36. doi:10.1590/2237-6089-2014-0031

19. Kin NM, Nair NP, Amin M, Schwartz G, Ahmed SK, Holm P, Katona C, Kragh-Sorensen P, Klitgaard N, Song WY, et al. The dexamethasone suppression test and treatment outcome in elderly depressed patients participating in a placebo-controlled multicenter trial involving moclobemide and nortriptyline. *Biol Psychiatry* (1997) **42**:925–31. Available at: http://www.ncbi.nlm.nih.gov/pubmed/9359979

20. Bschor T, Ritter D, Winkelmann P, Erbe S, Uhr M, Ising M, Lewitzka U. Lithium monotherapy increases ACTH and cortisol response in the DEX/CRH test in unipolar depressed subjects. A study with 30 treatment-naive patients. *PLoS One* (2011) **6**:e27613. doi:10.1371/journal.pone.0027613

21. Bschor T, Baethge C, Adli M, Eichmann U, Ising M, Uhr M, Müller-Oerlinghausen B, Bauer M. Lithium augmentation increases post-dexamethasone cortisol in the dexamethasone suppression test in unipolar major depression. *Depress Anxiety* (2003) **17**:43–48. doi:10.1002/da.10078

22. Bschor T, Adli M, Baethge C, Eichmann U, Ising M, Uhr M, Modell S, Künzel H, Müller-Oerlinghausen B, Bauer M. Lithium augmentation increases the ACTH and cortisol response in the combined DEX/CRH test in unipolar major depression. *Neuropsychopharmacology* (2002) **27**:470–478. doi:10.1016/S0893-133X(02)00323-8

23. B.S. M, S. A, M. G, T. K, B. K, G.S. A. State specificity of DST abnormalities in geriatric depression. *Biol Psychiatry* (1993) **34**:108–114. Available at: http://www.embase.com/search/results?subaction=viewrecord&from=export&id=L23240029%5Cnhttp://sfx.library.uu.nl/utrecht?sid=EMBASE&issn=00063223&id=doi:&atitle=State+specificity+of+DST+abnormalities+in+geriatric+depression&stitle=BIOL.+PSYCHIATRY&title=Bio

24. Horstmann S, Dose T, Lucae S, Kloiber S, Menke A, Hennings J, Spieler D, Uhr M, Holsboer F, Ising M. Suppressive effect of mirtazapine on the HPA system in acutely depressed women seems to be transient and not related to antidepressant action. *Psychoneuroendocrinology* (2009) **34**:238–248. doi:10.1016/j.psyneuen.2008.09.004

25. Amsterdam JD, Maislin G, Skolnick B, Berwish N, Winikur A. Multiple hormone response to clonidine administration in depressed patients and healthy volunteers. *Biol Psychiatry* (1989) **26**:265–278.

26. Ninan PT, Shelton RC, Bao W, Guico-Pabia CJ. BDNF, interleukin-6, and salivary cortisol levels in depressed patients treated with desvenlafaxine. *Prog Neuro-Psychopharmacology Biol Psychiatry* (2014) **48**:86–91. doi:10.1016/j.pnpbp.2013.09.016

27. Wang Y, Jia Y, Chen X, Ling X, Liu S, Xu G, Huang L. Hippocampal N-acetylaspartate and morning cortisol levels in drug-naive, first-episode patients with major depressive disorder: Effects of treatment. *J Psychopharmacol* (2012) **26**:1463–1470. doi:10.1177/0269881112450781

28. Daffner-Bugía C, Laakmann G, Voderholzer U, Haag C, Baghai T, Kolmsee S, Schröder U, Munz T. The neuroendocrine effects of venlafaxine in healthy subjects. *Hum Psychopharmacol* (1996) **11**:1–9. doi:10.1002/(SICI)1099-1077(199601)11:1<1::AID-HUP732>3.3.CO;2-Q

29. Scharnholz B, Weber-Hamann B, Lederbogen F, Schilling C, Gilles M, Onken V, Frankhauser P, Kopf D, Deuschle M. Antidepressant treatment with mirtazapine, but not venlafaxine, lowers cortisol concentrations in saliva: A randomised open trial. *Psychiatry Res* (2010) **177**:109–113. doi:10.1016/j.psychres.2009.08.010

30. Papakostas YG, Markianos M, Zervas IM, Theodoropoulou M, Vaidakis N, Daras M. Administration of citalopram before ECT: Seizure duration and hormone responses. *J ECT* (2000) **16**:356–360. doi:10.1097/00124509-200012000-00005

31. Hinkelmann K, Moritz S, Botzenhardt J, Muhtz C, Wiedemann K, Kellner M, Otte C. Changes in cortisol secretion during antidepressive treatment and cognitive improvement in patients with major depression: A longitudinal study. *Psychoneuroendocrinology* (2012) **37**:685–692. doi:10.1016/j.psyneuen.2011.08.012

32. Thakore JH, Barnes C, Joyce J, Medbak S, Dinan TG. Effects of antidepressant treatment on corticotropin-induced cortisol responses in patients with melancholic depression. *Psychiatry Res* (1997) **73**:27–32. doi:10.1016/S0165-1781(97)00106-6

33. Piwowarska J, Chimiak A, Matsumoto H, Dziklińska A, Radziwoń-Zaleska M, Szelenberger W, Pachecka J. Serum cortisol concentration in patients with major depression after treatment with fluoxetine. *Psychiatry Res* (2012) **198**:407–411. doi:10.1016/j.psychres.2012.01.029

34. Kauffman RP, Castracane VD, White DL, Baldock SD, Owens R. Impact of the selective serotonin reuptake inhibitor citalopram on insulin sensitivity, leptin and basal cortisol secretion in depressed and non-depressed euglycemic women of reproductive age. *Gynecol Endocrinol* (2005) **21**:129–137. doi:10.1080/09513590500216800

35. Vythilingam M, Vermetten E, Anderson GM, Luckenbaugh D, Anderson ER, Snow J, Staib LH, Charney DS, Bremner JD. Hippocampal volume, memory, and cortisol status in major depressive disorder: Effects of treatment. *Biol Psychiatry* (2004) **56**:101–112. doi:10.1016/j.biopsych.2004.04.002

36. Bschor T, Ising M, Erbe S, Winkelmann P, Ritter D, Uhr M, Lewitzka U. Impact of citalopram on the HPA system. A study of the combined DEX/CRH test in 30 unipolar depressed patients. *J Psychiatr Res* (2012) **46**:111–117. doi:10.1016/j.jpsychires.2011.09.020

37. Nikisch G, Mathé AA, Czernik A, Thiele J, Bohner J, Eap CB, Ågren H, Baumann P. Long-term citalopram administration reduces responsiveness of HPA axis in patients with major depression: Relationship with S-citalopram concentrations in plasma and cerebrospinal fluid (CSF) and clinical response. *Psychopharmacology (Berl)* (2005) **181**:751–760. doi:10.1007/s00213-005-0034-3

38. Zobel AW, Schulze-Rauschenbach S, Von Widdern OC, Metten M, Freymann N, Grasmäder K, Pfeiffer U, Schnell S, Wagner M, Maier W. Improvement of working but not declarative memory is correlated with HPA normalization during antidepressant treatment. *J Psychiatr Res* (2004) **38**:377–383. doi:10.1016/j.jpsychires.2003.12.002

39. Ruhé HG, Khoenkhoen SJ, Ottenhof KW, Koeter MW, Mocking RJT, Schene AH. Longitudinal effects of the SSRI paroxetine on salivary cortisol in Major Depressive Disorder. *Psychoneuroendocrinology* (2015) **52**:261–271. doi:10.1016/j.psyneuen.2014.10.024

40. Šagud M, Pivac N, Mück-Šeler D, Jakovljević M, Mihaljević-Peleš A, Koršić M. Effects of Sertraline Treatment on Plasma Cortisol, Prolactin and Thyroid Hormones in Female Depressed Patients. *Neuropsychobiology* (2002) **45**:139–143. doi:10.1159/000054954

41. Jazayeri S, Keshavarz SA, Tehrani-Doost M, Djalali M, Hosseini M, Amini H, Chamari M, Djazayery A. Effects of eicosapentaenoic acid and fluoxetine on plasma cortisol, serum interleukin-1beta and interleukin-6 concentrations in patients with major depressive disorder. *Psychiatry Res* (2010) **178**:112–115. doi:10.1016/j.psychres.2009.04.013

42. Seifritz E, Baumann P, Müller MJ, Annen O, Amey M, Hemmeter U, Hatzinger M, Chardon F, Holsboer-Trachsler E. Neuroendocrine effects of a 20-mg citalopram infusion in healthy males a placebo-controlled evaluation of citalopram as 5-HT function probe. *Neuropsychopharmacology* (1996) **14**:253–263. doi:10.1016/0893-133X(95)00117-V

43. Demisch K, Demisch L, Bochnik H, Nickelsen T, Althoff P, Schoffling K, Rieth R. Melatonin and cortisol increase after fluvoxamine [letter]. *Br J Clin Pharmacol* (1986) **22**:620–622. doi:10.1111/j.1365-2125.1986.tb02947.x

44. Sarubin N, Nothdurfter C, Schmotz C, Wimmer AM, Trummer J, Lieb M, Uhr M, Baghai TC, Wetter TC, Bühner M, et al. Impact on cortisol and antidepressant efficacy of quetiapine and escitalopram in depression. *Psychoneuroendocrinology* (2014) **39**:141–151. doi:10.1016/j.psyneuen.2013.10.008

45. Nothdurfter C, Schmotz C, Sarubin N, Baghai TC, Laenger A, Lieb M, Bondy B, Rupprecht R, Schüle C. Effects of escitalopram/quetiapine combination therapy versus escitalopram monotherapy on hypothalamic-pituitary-adrenal-axis activity in relation to antidepressant effectiveness. *J Psychiatr Res* (2014) **52**:15–20. doi:10.1016/j.jpsychires.2014.01.013

46. Straneva-Meuse PA, Light KC, Allen MT, Golding M, Girdler SS. Bupropion and paroxetine differentially influence cardiovascular and neuroendocrine responses to stress in depressed patients. *J Affect Disord* (2004) **79**:51–61. doi:10.1016/S0165-0327(02)00352-X

47. Nickel T, Sonntag A, Schill J, Zobel AW, Ackl N, Brunnauer A, Murck H, Ising M, Yassouridis A, Steiger A, et al. Clinical and neurobiological effects of tianeptine and paroxetine in major depression. *J Clin Psychopharmacol* (2003) **23**:155–168. doi:10.1097/00004714-200304000-00008

48. Deuschle M, Hamann B, Meichel C, Krumm B, Lederbogen F, Kniest A, Colla M, Heuser I. Antidepressive treatment with amitriptyline and paroxetine: Effects on saliva cortisol concentrations. *J Clin Psychopharmacol* (2003) **23**:201–205. doi:10.1097/00004714-200304000-00014

49. Mück-Šeler D, Pivac N, Šagud M, Jakovljevi M, Mihaljevi-Peleš A. The effects of paroxetine and tianeptine on peripheral biochemical markers in major depression. *Prog Neuro-Psychopharmacology Biol Psychiatry* (2002) **26**:1235–1243. doi:10.1016/S0278-5846(02)00259-2

50. Weber-Hamann B, Kratzsch J, Kopf D, Lederbogen F, Gilles M, Heuser I, Deuschle M. Resistin and adiponectin in major depression: The association with free cortisol and effects of antidepressant treatment. *J Psychiatr Res* (2007) **41**:344–350. doi:10.1016/j.jpsychires.2006.01.002

51. Manthey L, Leeds C, Giltay EJ, van Veen T, Vreeburg SA, Penninx BWJH, Zitman FG. Antidepressant use and salivary cortisol in depressive and anxiety disorders. *Eur Neuropsychopharmacol* (2011) **21**:691–699. doi:10.1016/j.euroneuro.2011.03.002

52. Rota E, Broda R, Cangemi L, Migliaretti G, Paccotti P, Rosso C, Torre E, Zeppegno P, Portaleone P. Neuroendocrine (HPA axis) and clinical correlates during fluvoxamine and amitriptyline treatment. *Psychiatry Res* (2005) **133**:281–284. doi:10.1016/j.psychres.2003.12.030

53. Kopf D, Westphal S, Luley CW, Ritter S, Gilles M, Weber-Hamann B, Lederbogen F, Lehnert H, Henn FA, Heuser I, et al. Lipid metabolism and insulin resistance in depressed patients: Significance of weight, hypercortisolism, and antidepressant treatment. *J Clin Psychopharmacol* (2004) **24**:527–531. doi:10.1097/01.jcp.0000138762.23482.63

54. Weber-Hamann B, Blum WF, Kratzsch J, Gilles M, Heuser I, Deuschle M. Insulin-like Growth Factor-I (IGF-I) serum concentrations in depressed patients: Relationship to saliva cortisol and changes during antidepressant treatment. *Pharmacopsychiatry* (2009) **42**:23–28. doi:10.1055/s-0028-1085442

55. Skene D, Bojkowski C, Arendt J. Comparison of the effects of acute fluvoxamine and desipramine administration on melatonin and cortisol production in humans. *Br J Clin Pharmacol* (1994) **37**:181–186. doi:10.1111/j.1365-2125.1994.tb04258.x

56. Sachar EJ, Hellman L, Roffwarg HP, Halpern FS, Fukush DK, Gallagher TF. Disrupted 24 hour patterns of cortisol secretion in psychotic depressives. *Arch Gen Psychiatry* (1973) **28**:19–24.

57. Heuser IJE, Schweiger U, Gotthard U, Schmider J, Lammers C-H, Dettling M, Yassouridis A, Holsboer F. Pituitary-adrenal-system regulation and psychopathology during amitriptyline treatment in elderly depressed patients and normal comparison subjects Heuser, Isabella J E; Schweiger, Ulrich; Gotthardt, Ulrike; Schmider, Jurgen; et al. The American Journal. *Am J Psychiatry* (1996) **153**:93–99.

58. Carson SW, Halbreich U. Effect of treatment on plasma cortisol and dexamethasone. *Biol Psychiatry* (1987) **22**:213–216. doi:10.1016/0006-3223(87)90233-2

59. Schmider J, Deuschle M, Schweiger U, Korner A, Gotthardt U, Heuser IJ. Amitriptyline metabolism in elderly depressed patients and normal controls in relation to hypothalamic-pituitary-adrenal system function. *J Clin Psychopharmacol* (1995) **15**:250–258. doi:10.1097/00004714-199508000-00003

60. O’Keane V, McLoughlin D, Dinan TG. D-fenfluramine-induced prolactin and cortisol release in major depression: Response to treatment. *J Affect Disord* (1992) **26**:143–150. doi:10.1016/0165-0327(92)90009-U

61. Lisansky J, Giovanni AFAVA, Zielezny MA, Morphy MA. Nocturnal prolactin and cortisol secretion and recovery from melancholia. *Psychoneuroendocrinology* (1987) **12**:303–311.

62. Piwowarska J, Wrzosek M, Radziwon-Zaleska M, Ryszewska-Pokrasniewicz B, Skalski M, Matsumoto H, Biernacka-Bazyluk A, Szelenberger W, Pachecka J. Serum cortisol concentration in patients with major depression after treatment with clomipramine. *Pharmacol Rep* (2009) **61**:604–611.

63. Shapira B, Yagmur MJ, Gropp C, Newman M, Lerer B. Effect of clomipramine and lithium on fenfluramine-induced hormone release in major depression. *Biol Psychiatry* (1992) **31**:975–983. doi:10.1016/0006-3223(92)90091-D

64. Coote M, Wilkins A, Werstiuk ES, Steiner M. Effects of electroconvulsive therapy and desipramine on neuroendocrine responses to the clonidine challenge test. *J Psychiatry Neurosci* (1998) **23**:172–178.

65. Sonntag A, Rothe B, Guldner J, Yassouridis A, Holsboer F, Steiger A. Trimipramine and imipramine exert different effects on the sleep EEG and on nocturnal hormone secretion during treatment of major depression. *Depression* (1996) **4**:1–13. doi:10.1002/(SICI)1522-7162(1996)4:1<1::AID-DEPR1>3.0.CO;2-S

66. Künzel HE, Ackl N, Hatzinger M, Held K, Holsboer-Trachsler E, Ising M, Kaschka W, Kasper S, Konstantinidis A, Sonntag A, et al. Outcome in delusional depression comparing trimipramine monotherapy with a combination of amitriptyline and haloperidol - A double-blind multicenter trial. *J Psychiatr Res* (2009) **43**:702–710. doi:10.1016/j.jpsychires.2008.10.004

67. Holsboer-Trachsler E, Stohler R, Hatzinger M. Repeated administration of the combined dexamethasone-human corticotropin releasing hormone stimulation test during treatment of depression. *Psychiatry Res* (1991) **38**:163–171.

68. Laakmann G, Wittmann M, Gugath M, Mueller OA, Treuschl J, Wahlster U, Staha GK. Effects of psychotropic drugs (desimipramine, chlorimipramine, sulpiride and diazepam) on the human HPA axis. *Psychopharmacology (Berl)* (1984) **84**:66–70. Available at: https://link.springer.com/content/pdf/10.1007%2FBF00432027.pdf

69. Linkowski P, Mendlewicz J, Kerkhofs M, Leclercq R, Golstein J, Brasseur M, Copinschi G, Cauter E Van. 24-Hour profiles of adrenocorticotropin, cortisol, and growth hormone in major depressive illness: Effect of antidepressant treatment. *J Clin Endocrinol Metab* (1987) **65**:141–152. doi:10.1210/jcem-65-1-141

70. Georgotas A, Stokes P, McCue RE, Dubow A, Welkowitz J, Friedman E, Fanelli C, Chang I, Cooper TB. The usefulness of DST in predicting response to antidepressants: A placebo-controlled study. *J Affect Disord* (1986) **11**:21–28. doi:10.1016/0165-0327(86)90055-8
