# Supplementary material for: Cortisol and Major Depressive Disorder—Translating Findings From Humans to Animal Models and Back
Source: Front Psychiatry. 2020 Jan 22;10:974. doi: 10.3389/fpsyt.2019.00974 (PMC6987444; doi:10.3389/fpsyt.2019.00974)
Supplement: Supplementary file 2 [file Table_2.docx]

**Supplementary Table 2:** Effects of treatment on corticosterone levels and behaviour in animal studies.

| **Antidepressant**  **Class** | **Drug** | **Dose** | **Duration** | **Stressed model** | **Basal CORT** | **Post-stress / DEX CORT** | **Behavioural effects** | **Reference** |
| --- | --- | --- | --- | --- | --- | --- | --- | --- |
| **SSRI/SNRI** | Fluoxetine | 10mg/kg | Single injection | None - Mice | ↑ |  | N/A | Weber et al., 2006 (1) |
|  | Fluoxetine | 10mg/kg | 3 injections | None - Rats |  | ↑ | Reduced immobility in FST | Rogóz et al., 2012 (2) |
|  | Fluoxetine | 10mg/kg | 3 injections | None - Rats | ↑ |  | Increased anxiety | Gomez et al., 2015 (3) |
|  | Fluoxetine | 10mg/kg | 2 weeks | None - Mice | ↑ |  | Restored hyperactivity and depressed behaviour induced by olfactory bulbectomy | Machado et al., 2012 (4) |
|  | Fluoxetine | 10mg/kg | 2 weeks | None - Mice | ↑ |  | N/A | Weber et al., 2006 (1) |
|  | Fluoxetine | 5mg/kg | 3 weeks | None - Rats | — |  | Increased immobility in FST | Mitic et al., 2013 (5) |
|  | Fluoxetine | 100 mg/L fluoxetine in water | 5 weeks | None - Mice | — |  | No effect on learned helplessness or immobility in FST | Tyler et al., 2014 (6) |
|  | Fluoxetine | 10mg/kg | 2 weeks | CUMS - Rats | ↓ |  | Restored stress-induced anhedonic and anxiety-like behaviour, and reduced immobility in FST | Cai et al., 2015 (7) |
|  | Fluoxetine | 10-15mg/kg (water) | 16 days | Repeated restraint stress -Mice |  | ↓ | Restored stress-induced anxiety-like behaviour | Ihne et al., 2012 (8) |
|  | Fluoxetine | 10mg/kg | 3 weeks | Prenatal stress - Rats | — | ↓ | Restored stress-induced anxiety-like behaviour and reduced immobility in FST | Szymańska et al., 2009 (9) |
|  | Fluoxetine | 15mg/kg | 3 weeks | CUMS - Rats | ↓ | Post-DEX: ↓ | Restored stress-induced anhedonic and anxiety-like behaviour, and reduced immobility in FST | Xing et al., 2015 (10) |
|  | Fluoxetine | 10mg/kg | 4 weeks | Social defeat stress -Mice | ↓ |  | Increased social interaction time | Wu et al., 2011 (11) |
|  | Fluoxetine | 10mg/kg | 6 weeks | CUMS - Rats | — |  | N/A | Zhang et al., 2010 (12) |
|  | Venlafaxine | 10mg/kg | 24 days | None - Rats | — | — | No effect on the immobility in FST | Connor et al., 2000 (13) |
|  | Venlafaxine | 10mg/kg | 21 days | CMS - Rats | ↓ |  | Restored stress-induced anhedonic behaviour | Xing et al., 2013 (14) |
|  | Venlafaxine | 7.81mg/kg | 27 days | CUMS - Rats | ↓ |  | Restored stress-induced anhedonic behaviour | Zhai et al., 2015 (15) |
|  | Venlafaxine | 30mg/kg | 6 weeks | CUMS - Rats | ↓ |  | N/A | Zhang et al., 2010 (12) |
| **TCA** | Amitriptyline | 10mg/kg | Single injection | None - Mice | — |  | N/A | Weber et al., 2006 (1) |
|  | Amitriptyline | 10mg/kg | 14 days | None - Mice | — |  | N/A | Weber et al., 2006 (1) |
|  | Desipramine | 10, 10, 20 mg/kg | Three injections | None - Mice | — | ↓ | Reduced immobility in FST and TST | Conti, 2004 (16) |
|  | Desipramine | 7.5mg/kg | 24 days | None - Rats | — | ↓ | Reduced immobility in FST | Connor et al., 2000 (13) |
|  | Desipramine | 10mg/kg | Twice daily for 21 days | None - Mice | — | ↓ | Reduced immobility in FST and TST | Conti, 2004 (16) |
|  | Desipramine | 10mg/kg | 14 days | Open field exposure - Rats |  | ↓ | Reduced anxiety-like behaviour | Wrona et al., 2013 (17) |
|  | Imipramine | 15mg/kg | Single injection | None - Mice |  | ↓ | Reduced immobility in FST and TST | Li et al., 2013 (18) |
|  | Imipramine | 30mg/kg | Single injection | None - Mice | ↑ |  | Reduced immobility in FST | Li et al., 2014 (19) |
|  | Imipramine | 10mg/kg | 14 days | None - Rats |  | — | N/A | Dazzi et al., 2001 (20) |
|  | Imipramine | 10mg/kg | 21 days | None - Rats | — |  | Reduced anxiety-like behaviour in novelty suppressed feeding test, while no effect on hedonic behaviour | Wainwright et al., 2016 (21) |
|  | Imipramine | 15mg/kg | 23 days | CUMS - Mice | ↓ |  | Restored stress-induced anhedonic behaviour and reduced immobility in FST | Dhingra and Bansal, 2015 (22) |
|  | Imipramine | 10mg/kg | 5 weeks | CMS - Mice | ↓ |  | Restored stress-induced anhedonic behaviour and reduced immobility in FST | Mizuki et al., 2014 (23) |
|  | Imipramine | 10mg/kg | 3 weeks | Prenatal stress - Rats | ↓ | ↓ | Restored stress-induced anxiety-like behaviour and reduced immobility in FST | Szymańska et al., 2009 (9) |
|  | Imipramine | 15mg/kg | 5 weeks | CUMS - Mice | ↓ |  | Restored stress-induced anhedonic behaviour | Li et al., 2013 (18) |
| **Atypical ADs** | Mirtazapine | 10mg/kg | Single injection | None - Mice | ↑ |  | N/A | Weber et al., 2006 (1) |
|  | Mirtazapine | 10mg/kg | 3 injections | None - Rats |  | — | No effect on immobility in FST | Rogóz et al., 2012 (2) |
|  | Mirtazapine | 10mg/kg | 14 days | None - Mice | — |  | N/A | Weber et al., 2006 (1) |
|  | Mirtazapine | 10mg/kg | 14 days | None - Rats |  | — | N/A | Dazzi et al., 2001 (20) |
|  | Mirtazapine | 10mg/kg | 3 weeks | Prenatal stress - Rats | ↓ | ↓ | Restored stress-induced anxiety-like behaviour and reduced immobility in FST | Szymańska et al., 2009 (9) |
|  | Mirtazapine | 5mg/kg | 6 weeks | CUMS - Rats | ↓ |  | N/A | Zhang et al., 2010 (12) |
|  | Risperidone | 0.1mg/kg or 0.05mg/kg | 3 injections | None - Rats |  | — | No effect on immobility in FST | Rogóz et al., 2012 (2) |
|  | Tianeptine | 10mg/kg | 3 weeks | Prenatal stress - Rats | — | ↓ | Restored stress-induced anxiety-like behaviour and reduced immobility in FST | Szymańska et al., 2009 (9) |
| **Lithium** | Lithium | 1.4mEq/kg | Single injection | None - Rats | ↑ |  | N/A | Vatal and Aiyar, 1983 (24) |
|  | Lithium | 45 meq of Li+ | > 6 days | None - Rats | ↑ |  | N/A | Vatal and Aiyar, 1983 (24) |
|  | Lithium | 1mg/kg | Single injection | Social isolation stress - Mice | — | — | No effect on depressive behaviour or anxiety-like behaviour | Haj-Mirzaian et al., 2016 (25) |
|  | Lithium | 10mg/kg | Single injection | Social isolation stress - Mice | — | ↓ | Restored depressive and anxiety-like behaviour | Haj-Mirzaian et al., 2016 (25) |
|  | Lithium | 2.5mEq/kg | 14 days | CMS - Rats | ↓ |  | Reduced immobility in FST | Silva et al., 2008 (26) |
| **ECS** | ECS |  | Single | None - Rats | ↑ |  | N/A | Thiagarajan et al., 1989 (27) |
|  | ECS | Pulse width 1.5 ms, 50 Hz, 0.5 s | 10 days | None - Rats | ↑ |  | N/A | Gur et al., 2002 (28) |
|  | ECS | 80 *μ*A, 60 Hz, 0.2 s | 10 days | None - Rats | ↑ |  | N/A | Herman et al., 1989 (29) |
|  | ECS | 55–65 mA, 60 Hz, 0.8 s | 5 days | WKY rats | — |  | Reduced immobility in FST and restored retrograde memory in WKY rats | Kyeremanteng et al., 2014 (30) |
|  | ECS | 100 V, 50 Hz, 1.5 s | 9 days | FSL rats | — |  | Reduced immobility in FST | Maayan et al., 2005 (31) |

^CUMS, Chronic unpredictable mild stress; CMS, Chronic mild stress; FST, Forced swim test; TST, Tail suspension test; — No effect; ↓ Reduction; ↑ Increase^

**References**

1. Weber C-C, Eckert GP, Müller WE. Effects of Antidepressants on the Brain/Plasma Distribution of Corticosterone. *Neuropsychopharmacology* (2006) **31**:2443–2448. doi:10.1038/sj.npp.1301076

2. Rogóz Z, Kabziński M, Sadaj W, Rachwalska P, Ga̧dek-Michalska A. Effect of co-treatment with fluoxetine or mirtazapine and risperidone on the active behaviors and plasma corticosterone concentration in rats subjected to the forced swim test. *Pharmacol Reports* (2012) **64**:1391–1399. doi:10.1016/S1734-1140(12)70936-2

3. Gomez F, Venero C, Viveros M-P, García-García L. Short-term fluoxetine treatment induces neuroendocrine and behavioral anxiogenic-like responses in adolescent male rats. *Exp Brain Res* (2015) **233**:983–995. doi:10.1007/s00221-014-4173-9

4. Machado DG, Cunha MP, Neis VB, Balen GO, Colla A, Grando J, Brocardo PS, Bettio LEB, Capra JC, Rodrigues ALS. Fluoxetine reverses depressive-like behaviors and increases hippocampal acetylcholinesterase activity induced by olfactory bulbectomy. *Pharmacol Biochem Behav* (2012) **103**:220–229. doi:10.1016/J.PBB.2012.08.024

5. Mitic M, Simic I, Djordjevic J, Radojcic MB, Adzic M. Gender-specific effects of fluoxetine on hippocampal glucocorticoid receptor phosphorylation and behavior in chronically stressed rats. *Neuropharmacology* (2013) **70**:100–111. doi:10.1016/J.NEUROPHARM.2012.12.012

6. Tyler CR, Solomon BR, Ulibarri AL, Allan AM. Fluoxetine treatment ameliorates depression induced by perinatal arsenic exposure via a neurogenic mechanism. *Neurotoxicology* (2014) **44**:98–109. doi:10.1016/J.NEURO.2014.06.006

7. Cai L, Li R, Tang W, Meng G, Hu X, Wu T. Antidepressant-like effect of geniposide on chronic unpredictable mild stress-induced depressive rats by regulating the hypothalamus–pituitary–adrenal axis. *Eur Neuropsychopharmacol* (2015) **25**:1332–1341. doi:10.1016/J.EURONEURO.2015.04.009

8. Ihne JL, Fitzgerald PJ, Hefner KR, Holmes A. Pharmacological modulation of stress-induced behavioral changes in the light/dark exploration test in male C57BL/6J mice. *Neuropharmacology* (2012) **62**:464–473. doi:10.1016/J.NEUROPHARM.2011.08.045

9. Szymańska M, Budziszewska B, Jaworska-Feil L, Basta-Kaim A, Kubera M, Leśkiewicz M, Regulska M, Lasoń W. The effect of antidepressant drugs on the HPA axis activity, glucocorticoid receptor level and FKBP51 concentration in prenatally stressed rats. *Psychoneuroendocrinology* (2009) **34**:822–832. doi:10.1016/J.PSYNEUEN.2008.12.012

10. Xing H, Zhang K, Zhang R, Shi H, Bi K, Chen X. Antidepressant-like effect of the water extract of the fixed combination of Gardenia jasminoides, Citrus aurantium and Magnolia officinalis in a rat model of chronic unpredictable mild stress. *Phytomedicine* (2015) **22**:1178–1185. doi:10.1016/J.PHYMED.2015.09.004

11. Wu J, Du J, Xu C, Le J, Xu Y, Liu B, Dong J. Icariin attenuates social defeat-induced down-regulation of glucocorticoid receptor in mice. *Pharmacol Biochem Behav* (2011) **98**:273–278. doi:10.1016/j.pbb.2011.01.008

12. Zhang Y, Gu F, Chen J, Dong W. Chronic antidepressant administration alleviates frontal and hippocampal BDNF deficits in CUMS rat. *Brain Res* (2010) **1366**:141–148. doi:10.1016/j.brainres.2010.09.095

13. Connor TJ, Kelliher P, Shen Y, Harkin A, Kelly JP, Leonard BE. Effect of Subchronic Antidepressant Treatments on Behavioral, Neurochemical, and Endocrine Changes in the Forced-Swim Test. *Pharmacol Biochem Behav* (2000) **65**:591–597. doi:10.1016/S0091-3057(99)00192-6

14. Xing Y, He J, Hou J, Lin F, Tian J, Kurihara H. Gender differences in CMS and the effects of antidepressant venlafaxine in rats. *Neurochem Int* (2013) **63**:570–575. doi:10.1016/j.neuint.2013.09.019

15. Zhai XJ, Chen F, Chen C, Zhu CR, Lu YN. LC-MS/MS based studies on the anti-depressant effect of hypericin in the chronic unpredictable mild stress rat model. *J Ethnopharmacol* (2015) **169**:363–369. doi:10.1016/j.jep.2015.04.053

16. Conti AC. Inducible cAMP Early Repressor Regulates Corticosterone Suppression after Tricyclic Antidepressant Treatment. *J Neurosci* (2004) **24**:1967–1975. doi:10.1523/JNEUROSCI.4804-03.2004

17. Wrona D, Listowska M, Kubera M, Majkutewicz I, Glac W, Wojtyła-Kuchta B, Plucińska K, Grembecka B, Podlacha M. Chronic antidepressant desipramine treatment increases open field-induced brain expression and spleen production of interleukin 10 in rats. *Brain Res Bull* (2013) **99**:117–131. doi:10.1016/J.BRAINRESBULL.2013.10.002

18. Li J, Geng D, Xu J, Weng L-J, Liu Q, Yi L-T. Antidepressant-like effect of macranthol isolated from Illicium dunnianum tutch in mice. *Eur J Pharmacol* (2013) **707**:112–119. doi:10.1016/J.EJPHAR.2013.03.010

19. Li B, Zhao J, Lv J, Tang F, liu L, Sun Z, Wang L, Siwela SP, Wang Y, Song Y, et al. Additive antidepressant-like effects of fasting with imipramine via modulation of 5-HT2 receptors in the mice. *Prog Neuro-Psychopharmacology Biol Psychiatry* (2014) **48**:199–206. doi:10.1016/J.PNPBP.2013.08.015

20. Dazzi L, Serra M, Spiga F, Pisu MG, Jentsch JD, Biggio G. Prevention of the stress-induced increase in frontal cortical dopamine efflux of freely moving rats by long-term treatment with antidepressant drugs. *Eur Neuropsychopharmacol* (2001) **11**:343–349. doi:10.1016/S0924-977X(01)00105-5

21. Wainwright SR, Workman JL, Tehrani A, Hamson DK, Chow C, Lieblich SE, Galea LAM. Testosterone has antidepressant-like efficacy and facilitates imipramine-induced neuroplasticity in male rats exposed to chronic unpredictable stress. *Horm Behav* (2016) **79**:58–69. doi:10.1016/j.yhbeh.2016.01.001

22. Dhingra D, Bansal S. Antidepressant-like activity of plumbagin in unstressed and stressed mice. *Pharmacol Reports* (2015) **67**:1024–1032. doi:10.1016/J.PHAREP.2015.03.001

23. Mizuki D, Matsumoto K, Tanaka K, Thi Le X, Fujiwara H, Ishikawa T, Higuchi Y. Antidepressant-like effect of Butea superba in mice exposed to chronic mild stress and its possible mechanism of action. *J Ethnopharmacol* (2014) **156**:16–25. doi:10.1016/J.JEP.2014.08.014

24. Vatal M, Aiyar AS. Some aspects of corticosterone metabolism in lithium treated rats. *Chem Biol Interact* (1983) **45**:277–282. doi:10.1016/0009-2797(83)90074-1

25. Haj-Mirzaian A, Amiri S, Kordjazy N, Momeny M, Razmi A, Rahimi-Balaei M, Amini-Khoei H, Haj-Mirzaian A, Marzban H, Mehr SE, et al. Lithium attenuated the depressant and anxiogenic effect of juvenile social stress through mitigating the negative impact of interlukin-1β and nitric oxide on hypothalamic-pituitary-adrenal axis function. *Neuroscience* (2016) **315**:271–285. doi:10.1016/j.neuroscience.2015.12.024

26. Silva R, Mesquita AR, Bessa J, Sousa JC, Sotiropoulos I, Leão P, Almeida OFX, Sousa N. Lithium blocks stress-induced changes in depressive-like behavior and hippocampal cell fate: The role of glycogen-synthase-kinase-3β. *Neuroscience* (2008) **152**:656–669. doi:10.1016/j.neuroscience.2007.12.026

27. Thiagarajan AB, Gleiter CH, Mefford IN, Eskay RL, Nutt DJ. Effect of single and repeated electroconvulsive shock on the hypothalamic-pituitary-adrenal axis and plasma catecholamines in rats. *Psychopharmacology (Berl)* (1989) **97**:548–52. Available at: http://www.ncbi.nlm.nih.gov/pubmed/2543014 [Accessed July 30, 2018]

28. Gur E, Dremencov E, Garcia F, Van de Kar LD, Lerer B, Newman ME. Functional effects of chronic electroconvulsive shock on serotonergic 5-HT1A and 5-HT1B receptor activity in rat hippocampus and hypothalamus. *Brain Res* (2002) **952**:52–60. doi:10.1016/S0006-8993(02)03193-1

29. Herman JP, Schäfer MK-H, Sladek CD, Day R, Young EA, Akil H, Watson SJ. Chronic electroconvulsive shock treatment elicits up-regulation of CRF and AVP mRNA in select populations of neuroendocrine neurons. *Brain Res* (1989) **501**:235–246. doi:10.1016/0006-8993(89)90641-0

30. Kyeremanteng C, MacKay JC, James JS, Kent P, Cayer C, Anisman H, Merali Z. Effects of electroconvulsive seizures on depression-related behavior, memory and neurochemical changes in Wistar and Wistar–Kyoto rats. *Prog Neuro-Psychopharmacology Biol Psychiatry* (2014) **54**:170–178. doi:10.1016/J.PNPBP.2014.05.012

31. Maayan R, Morad O, Dorfman P, Overstreet DH, Weizman A, Yadid G. The involvement of dehydroepiandrosterone (DHEA) and its sulfate ester (DHEAS) in blocking the therapeutic effect of electroconvulsive shocks in an animal model of depression. *Eur Neuropsychopharmacol* (2005) **15**:253–262. doi:10.1016/J.EURONEURO.2004.10.005
